# Minimum detectable acuity: Diagnosis of colour deficiency and improvement with colour correcting lenses

**DOI:** 10.1038/s41433-025-04204-3

**Published:** 2026-01-03

**Authors:** Jeff Rabin, Cara Duka, Alyssa Hood, Katelyn Goodroe, Kyle Dunmon, Darien Bouaphavong, Thinh Truong

**Affiliations:** University of the Incarnate Word Rosenberg School of Optometry, San Antonio, TX USA

**Keywords:** Medical research, Business and industry

Hereditary colour vision deficiency (CVD) is a non-progressive X-linked condition (8% males, 1/200 females) due to a shift in peak sensitivity of red (long wavelength, L) or green (middle wavelength, M) cones or the absence of either cone photopigments [1]. Minimum visible (detectable) acuity (MDA) is the thinnest black line detectable on a white background but heretofore has not been used clinically. Recently we developed a cone-specific MDA test [2]. We extended this research to determine if cone-specific MDA: (a) diagnoses type and severity of hereditary CVD (b) improves with colour vision correcting lenses (CCLs)[3, 4].

**Fig. 1:**
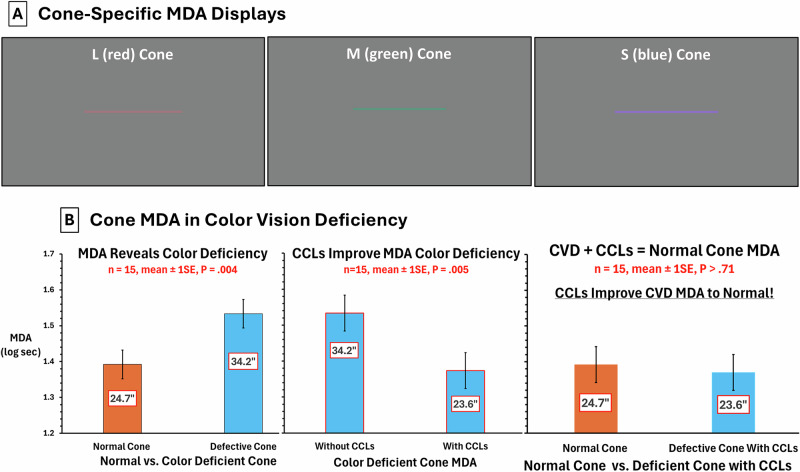
MDA Displays and Cone-Specific MDA in Colour Vision Deficiency. **A** MVA Displays. MDA stimuli are shown for L cone, M cone and S cone stimuli. On each trial a single line appeared (specific colour and orientation). Subjects reported both orientation and colour across a range of line thicknesses. **B** Cone MDA in Colour Vision Deficiency. Left plot: increase in MDA threshold (poorer performance) for deficient vs. normal cone (*P* = 0.004). Middle plot: better deficient cone MDA with CCLs (*P* = 0.005). Right plot: *no difference* between normal and deficient cone when wearing CCLs (*P* > 0.71).

Fifteen CVDs (confirmed by Ishihara, cone contrast test [1], age 26 ± 8, 13 green, 2 red CVDs) participated after written informed consent (IRB approved protocol). MDA stimuli: 1.4° vertical, horizontal, or oblique lines centred on a Microsoft Surface display (3.7° x 2.1°, 4 m, dark room). Each line was an increase in L, M, or S stimulation against a grey background (24.7 cd/m^2^, *x,y* = 0.32,.36) converted to cone contrast [1] (L, M: 16%, S: 128% due to paucity of S cones, Fig. 1A). Line thickness varied from 60” to 10” of arc in 0.16 log steps. On each trial a single line appeared on the display. Subjects reported colour and orientation (0.01 log units/trial) randomised across trials. Log MDA thresholds were distributed normally for CVDs (*P* > 0.51). Paired t-tests with Bonferroni correction, Bland Altman and regression were used.

Figure 1B shows mean MDA threshold for the abnormal cone type (1.53 log sec, 34.15”) was significantly higher (poorer performance) than mean MDA for the normal cone type (1.39 log sec, 24.66”, mean difference 0.14 log sec, 9.49”, 95% CI 0.05–0.23, *P* = 0.004). Mean MDA threshold for the abnormal cone type without CCLs (1.53 log sec, 34.15”) was significantly higher than with CCLs (1.37 log sec, 23.62”, mean difference 0.16 log CS, 10.53”, 95% CI 0.06–0.26, *P* = 0.005). Yet there was no difference between MDA for the normal cone type compared to defective cone type with CCLs suggesting comparable performance (*P* > 0.71).

**Fig. 2:**
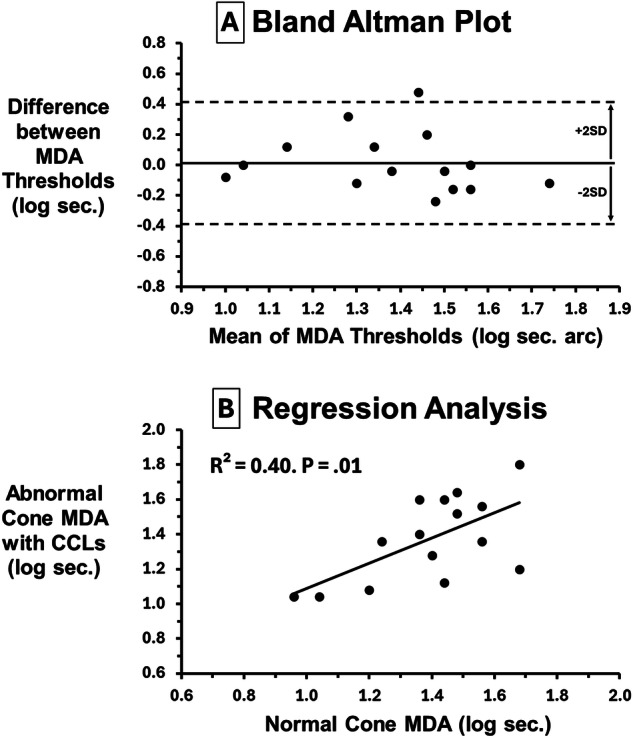
Bland Altman and Regression Analysis. **A** Bland Altman Plot. Since there was no difference between normal cone and defective cone MDA when wearing CCLs (*P* > 0.71), Fig. 2A shows a Bland Altman plot to assess MDA agreement and bias. The difference between normal and defective cone MDA with CCLs is plotted against their means. Mean difference is essentially zero (bold horizontal line = 0.02) indicating no bias. All but one difference falls within 95% CI ( ± 2 SD) of the mean without systematic changes indicating good agreement for this limited sample size. **B** Regression Analysis. Figure 2B shows how well normal cone performance predicts anomalous cone performance when wearing CCLs. Regression analysis (Fig. 2B) shows a highly significant prediction with the slope approaching unity (r^2^ = 0.40, F = 8.52, *P* = 0.01). This substantiates efficacy of CCLs to render abnormal cone performance comparable to that achieved by normal cones.

To assess MDA bias and agreement between normal cone and defective cone with CCLs, Fig. 2A shows Bland Altman analysis: normal cone – defective cone with CCLs plotted against means. Mean difference is zero (0.02) indicating no bias and most values fall within the 95% CI. To determine whether normal cone MDA predicts abnormal cone MDA when wearing CCLs, regression analysis (Fig. 2B) shows a highly significant prediction with the slope approaching unity (r^2^ = 0.40, F = 8.52, *P* = 0.01).

Limitations include small sample size and possible fatigue. Orientation and binocular specificity of MDA [2] support a cortical basis for this hyperacuity test. The MDA equivalence of normal cones and defective cones with CCLs exemplifies efficacy. CCL extended wear improves colour vision even without CCLs in place [3, 4] suggesting cortical gain and/or neuro-adaptive change from perceptual learning [5] making MDA optimal for assessing notch-filter CCLs and related interventions. Abbreviated cone-specific MDA testing shows potential for occupational assessment of hereditary CVD and detection of acquired CVD.
